# Improving HIV proteome annotation: new features of BioAfrica HIV Proteomics Resource

**DOI:** 10.1093/database/baw045

**Published:** 2016-04-16

**Authors:** Megan Druce, Chantal Hulo, Patrick Masson, Paula Sommer, Ioannis Xenarios, Philippe Le Mercier, Tulio De Oliveira

**Affiliations:** ^1^Africa Centre for Population Health, School of Laboratory Medicine and Medical Sciences, Nelson R. Mandela School of Medicine, College of Health Sciences, University of KwaZulu-Natal, Durban, South Africa; ^2^Division of Genetics, School of Life Sciences, University of KwaZulu-Natal, Durban, South Africa; ^3^Swiss-Prot Group, SIB Swiss Institute of Bioinformatics, Geneva, Switzerland

## Abstract

The Human Immunodeficiency Virus (HIV) is one of the pathogens that cause the greatest global concern, with approximately 35 million people currently infected with HIV. Extensive HIV research has been performed, generating a large amount of HIV and host genomic data. However, no effective vaccine that protects the host from HIV infection is available and HIV is still spreading at an alarming rate, despite effective antiretroviral (ARV) treatment. In order to develop effective therapies, we need to expand our knowledge of the interaction between HIV and host proteins. In contrast to virus proteins, which often rapidly evolve drug resistance mutations, the host proteins are essentially invariant within all humans. Thus, if we can identify the host proteins needed for virus replication, such as those involved in transporting viral proteins to the cell surface, we have a chance of interrupting viral replication. There is no proteome resource that summarizes this interaction, making research on this subject a difficult enterprise. In order to fill this gap in knowledge, we curated a resource presents detailed annotation on the interaction between the HIV proteome and host proteins. Our resource was produced in collaboration with ViralZone and used manual curation techniques developed by UniProtKB/Swiss-Prot. Our new website also used previous annotations of the BioAfrica HIV-1 Proteome Resource, which has been accessed by approximately 10 000 unique users a year since its inception in 2005. The novel features include a dedicated new page for each HIV protein, a graphic display of its function and a section on its interaction with host proteins. Our new webpages also add information on the genomic location of each HIV protein and the position of ARV drug resistance mutations. Our improved BioAfrica HIV-1 Proteome Resource fills a gap in the current knowledge of biocuration.

Database URL: http://www.bioafrica.net/proteomics/HIVproteome.html

## Introduction

The objective of the BioAfrica HIV-1 Proteomics Resource is to provide accurate and comprehensive information on Human Immunodeficiency Virus (HIV) proteins. The first version of the resource was published in 2005 (1) and became popular across the scientific community. But, as with any resource, there is a need for information to be current and accurate. This is especially true in the fields of bioinformatics and proteomics, which have recently seen a massive increase in knowledge of both HIV-1 and host proteins as well on antiretroviral (ARV) drugs used for HIV treatment. In order to produce an accurate and comprehensive resource that complements other online protein resources, Bioafrica started to collaborate with Swiss-Prot ViralZone group of the Swiss Institute of Bioinformatics (SIB).

The collaboration of ViralZone and BioAfrica was funded by the Swiss South Africa Joint Research Programme (SSAJRP) in order to allow much of the knowledge produced by their independent projects to be synergized and presented in a new online section of the Swiss and South Africa academic websites. One of our main goals was to add information on ARV drug resistance and to generate new knowledge of the pathogen and host interaction. We also aimed to represent current understanding in an innovative and interactive way with graphical images and with links to relevant protein databases. We applied biocuration methods developed by UniProtKB/Swiss-Prot, which included manual extraction and structuring of information from the literature, manual verification of results from computational analyses, and mining and integration of large-scale datasets. The resource now includes protein structure and function, gene expression, post-transcriptional and translational modification, protease cleavage sites, drug resistance information and HIV and host protein interactions. In this biocuration paper, we present the upgrade of the BioAfrica HIV Proteome Resource and its synergetic interaction with ViralZone/SIB.

## Methods

The original BioAfrica HIV-1 Proteome Resource and ViralZone were used as a starting point for the biocuration process. A team of researchers from South Africa and Switzerland updated the information using manual curation in accordance with Swiss-Prot standards (2). This involved reading a large number of abstracts, identifying relevant publications by searching literature databases and reviewing related UniProt protein pages. Papers were read in full and important information was extracted and summarized as text and images. We also manually accessed information in protein databases. Our updated resource provides a webpage for each of the 22 HIV-1 proteins. Each webpage is divided into six sections. These are: (i) General Overview, (ii) Protein Function and Host–Virus Protein Interactions, (iii) Genomic Location and Protein Sequence, (iv) Protein Domains/Folds/Motifs, (v) HIV ARVs and Drug Resistance Mutations and (vi) Primary and Secondary Database Entries. All of the proteome pages end with a list of the referenced articles, which are linked to PubMed. Below we describe the methods used for each section.

(i) The General Overview contains information on the main function and localization of the protein in the HIV-1 replication cycle. This section describes: (a) the main function of the protein, (b) protein isoforms, (c) protein cleavage sites and when these exist, (d) localization of the protein within the virus and host cell and (e) additional information on protein function. The objective of this section is to allow users to access key information on the function of each HIV-1 protein as well as on key online resources. This was the last section to be curated as it summarizes information presented in the other sections of the webpage. It also provides key links to other online resources such as ViralZone (2) HIV replication cycle, the Protein Database (PDB), Uniprot and the GenBank.

(ii) The section on Protein Function and Host–Virus Protein Interactions was produced from a critical literature review of experimental and predicted host protein interaction for each HIV-1 protein. All interactions were manually verified for each protein sequence in UniProtKB. The interactions were summarized in graphical images that were created in Illustrator, following the standard process in ViralZone (2). One of the objectives was to create images that represented the function of the proteins and that used standard colours and shapes. Much of the work summarized in the images was produced by deep annotation made by the ViralZone group alone. The images are used on the ViralZone and BioAfrica websites. The dual display of this information allows synergy to be produced between the websites and information to be consistent across sites.

(iii) The Genomic Location and Protein Sequence section presents the genomic coordinates and amino acid sequence for each HIV-1 protein. The genomic location is presented as a graphical image and was produced by Rega Subtyping Tool Version 3.0 (3). The graphical image and numbering positioning are produced according to HIV-1 reference sequence (i.e. HXB2). The amino acid sequence of the protein is displayed and can be downloaded as FASTA sequence.

(iv) The Protein Domains/Folds/Motifs section contains information from the analysis of the amino acid sequences of the reference protein by InterPro (4) and Prosite (5). InterPro is a freely available database that is used to classify sequences into protein families and to predict the presence of important domains and sites. The link provided to InterPro contains information on the biological process and molecular function of the main domain in the protein. For example, for HIV-1 protease, our resource links to the aspartic peptidase active site entry (IPR001969), which is a wide family of proteolytic enzymes known to exist in vertebrates, fungi, plants and retroviruses. The Prosite motifs of high probability of occurrence that are excluded from Interpro are also presented in the resource, such as *N*-glycosylation, *N*-myristoylation and protein kinase C phosphorylation site motifs.

(v) The HIV ARVs and Drug Resistance Mutations section is presented for the HIV-1 proteins that are targeted by ARVs. The section links information housed in ViralZone (2) and the Stanford HIV Drug Resistance Database (Stanford HIVDB) (6). The Stanford HIVDB contains detailed information on all of the licensed HIV drugs and how they interact with the HIV proteins (http://hivdb.stanford.edu). HIVDB also regularly provides an updated, in-depth, referenced summary of HIV drug resistance mutations. This is displayed in tables, with links to detailed information on how the mutations act on the HIV proteins in order to cause resistance. (http://bioafrica.mrc.ac.za/hivdb/pages/download/resistanceMutations_handout.pdf). This section contains a summary of the mechanism of action of the ARVs, which is followed by a table that highlights the drug resistance mutations and the ARVs affected. It was curated in collaboration with Stanford University HIV Drug Resistance Database (Stanford HIVdb) and links back to the respective pages on the Stanford HIVDB (6). We expect to continually upgrade the mutations and ARVs lists as we currently collaborate with Stanford on the maintenance of the southern African Stanford HIVDB mirror (7).

(vi) The Primary and Secondary Database Entries section provides links to related databases within each protein page of the HIV-1 Proteome Resource. All of the links are provided to the HIV-1 reference genome (HXB2). We have selected 15 well-curated databases to link to, including, among others, PDB/MMDB (http://www.ncbi.nlm.nih.gov/structure), Uniprot (http://www.uniprot.org/), EMBL-EBI (http://www.ebi.ac.uk/Tools/dbfetch/), InterPro (http://www.ebi.ac.uk/interpro/), Pfam (http://pfam.xfam.org/), SCOP (http://supfam.org/SUPERFAMILY/index.html), BLOCKS (http://blocks.fhcrc.org/), Prosite (http://prosite.expasy.org/), ProtoNet (http://www.protonet.cs.huji.ac.il/index.php), ModBase (http://modbase.compbio.ucsf.edu/modbase-cgi/index.cgi) and the HIV-1 Human Protein Interaction DB at NCBI (http://www.ncbi.nlm.nih.gov/gene/155971).

**Figure 1.**
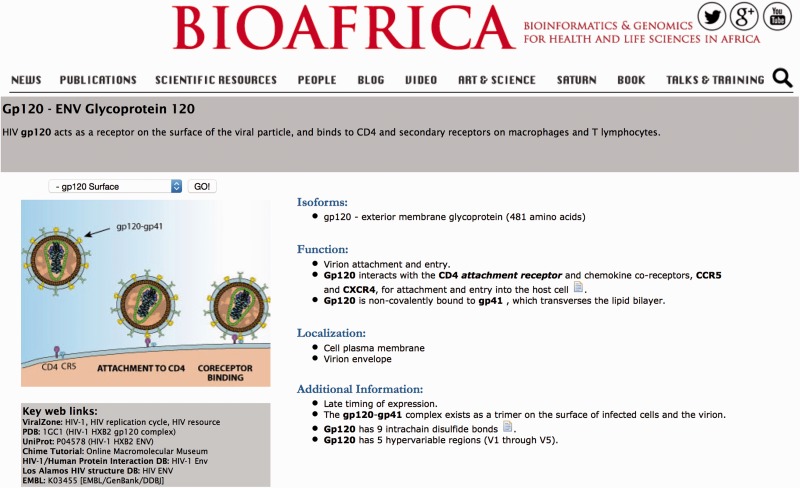
The general overview section of the BioAfrica HIV-1 Proteome Resource, as shown by the gp120 protein.

All of the proteome pages end with a list of the referenced articles, which are linked to PubMed. All of the links provided are functional and a script has been developed to check them every quarter. All of the updated webpages in the resource kept the original Bioafrica HIV proteomics resource html links. The reason for that is that these webpages also receive many links. For example, all of the 22 webpages of the resource are cross-linked from Uniprot in their HIV-1 reference sequence (i.e. HXB2) annotation page (e.g. http://www.uniprot.org/uniprot/P04608). The BioAfrica Proteome Resource is a part of http://www.bioafrica.net website. All pages from the previous version of the BioAfrica Proteome Resource are still online. However, we modified the html address to ‘proteomics2005’ (e.g. http://www.bioafrica.net//proteomics2005/POL-PRprot.html). This allows a comparison between the old and the newly upgraded resource (e.g. http://www.bioafrica.net/proteomics/POL-PRprot.html). In addition, Supplem entary Figure 1 shows the old and upgraded protease pages.

## Results

Our results section starts by presenting information on the HIV-1 proteome (i.e. all HIV-1 proteins) and their interaction with host–proteins. Each protein is also described in detail in the six sections of the manuscript, which mimic the sections of our online resource. We use the Env gp120/gp41 and Rev annotation pages as examples to present some of the results of the biocuration process. Other proteins pages are available at http://www.bioafrica.net/proteomics/HIVproteome.html.

The HIV-1 genome is approximately 9.75 kb in length. It codes for only nine open reading frames (ORFs), including the Gag, Pol, Env, Tat, Rev, Nef, Vif, Vpr and Vpu genes. The HIV-1 genome is capable of producing 19 proteins via alternative splicing, alternative translation, ribosomal frameshift, alternative initiation and post-translational cleavages (8). Within the BioAfrica resource, there are 22 webpages dedicated to the 19 HIV-1 proteins and to the 3 polyproteins.

### (i) General overview

Each HIV-1 protein page starts with a general overview. For example, the main function of the gp120 protein is viral attachment. This protein is an external membrane glycoprotein. It is localized at the host cell plasma membrane and virion envelope (more info at: http://bioafrica.net/proteomics/ENV-GP120prot.html and Figure 1). This section also contains a representative illustration of the protein in question. It also include a list of links to key online resources such as ViralZone, the Protein Database (PDB), Uniprot, the HIV-1/Human Protein Interaction Database, the Los Alamos HIV Sequence Database and EMBL/GenBank/DDBJ links.

### (ii) Protein function and host–virus protein interactions

**Figure 2.**
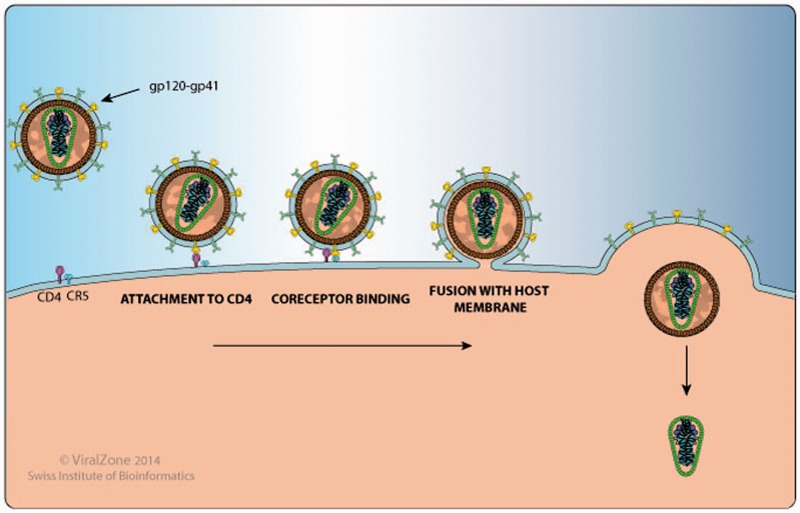
HIV-1 Gp120 and Gp41 protein interaction with human proteins CD4 and CCR5 , as shown by the Env protein attachment, co-receptor binding and fusion image.

This section includes a detailed and well-annotated image illustrating the function of the protein in question within the host cell and its host protein interactions. For example, on the Env gp120 proteome webpage (http://bioafrica.net/proteomics/ENV-GP120prot.html and Figure 2), the human proteins CD4, CCR5 and CXCR4, which are HIV-1 entry receptors found at the cell membrane are listed in the illustration and a link is provided to their Uniprot page. The gp120 webpage also links to a ViralZone webpage that describes in more detail the process of viral attachment to the host cell (http://viralzone.expasy.org/all_by_protein/3942.html). In addition, gp120 has been shown to interact with DC-SIGN/CD209 on the surface of dendritic cells to enhance virion transmission and infection. DC-SIGN also facilitates mucosal transmission by transporting HIV to lymphoid tissue (9,10).

Using the Rev protein page (http://bioafrica.net/proteomics/REVprot.html) as a second example, we show the Rev-mediated export of unspliced or incompletely spliced viral RNA transcripts from the host nucleus to the cytoplasm, facilitated by various Rev-host protein interactions (11–13). Host–virus protein interactions highlighted in the image at the webpage, include CRM1/XPO1, Importin-beta 1, B23, DDX3X and Sam68. Importin-beta 1 (14) and B23 (15) form a complex with RanGTP and Rev to facilitate the transport of Rev from the host cell cytoplasm to the nucleus. Once inside the host nucleus, DDX1 binds to Rev and the Rev-responsive element (RRE) to facilitate their transport within the cell nucleus (16). Following this CRM1, the Rev-RRE nuclear export receptor is bound by RanGTP to form a CRM1-RanGTP complex. This induces the formation of a Rev-RRE-CRM1-RanGTP complex and initiates the export of Rev-RRE out of the nucleus (17). DDX3 (18) and Sam68 (19) bind to this complex and enhance the Rev-mediated nuclear export of viral RNA. Further host-Rev protein interactions not highlighted in the image include DDX5 and DDX24. It has been proposed that the Rev-DDX5 interaction plays a role in HIV-1 replication and association interference could result in the reduction of viral replication (20).

In addition, all host and virus proteins have been linked to their appropriate UniProt (http://www.uniprot.org/) pages. Together, the Protein Function and Host–Virus Protein Interactions section provides users with an illustrated description of the role of the HIV-1 protein within the virus life cycle as well as descriptions of host–virus protein interactions linked to relevant publications and resources. All interactions listed have been proved by dozens of experiments, which were manually curated from the literature. Table 1 summarizes information on the host protein interactions for all HIV-1 proteins.

**Table 1.** Summarizes information on the main function of the proteins, their location on the host cell and host–virus protein interactions

HIV protein	Main function	Location	Host protein interaction	References
Env	Precursor of gp120 and gp41	ER and Golgi		
Gp120	Virion attachment	Cell plasma membrane and virion envelope	CD4, CCR5, CXCR4, DC-SIGN/CD209	(21–24)
Gp41	Mediates the fusion of viral and cellular membranes	Cell plasma membrane and virion envelope		
Gag	Mediates essential virion assembly and budding events	Cell plasma membrane, virion, host cytoplasm and late endosome/multivesicular bodies	ESCRT system, various interactions specific to each Gag protein product	(25)
P17 (Matrix)	Mediates virion assembly by targeting Gag and polyproteins to the plasma membrane and incorporates Env into budding virions	Inner surface of virion lipid bilayer, host cell cytoplasm, cell nucleus, late endosomes/multivesicular bodies and plasma membrane	Calmodulin (CaM), AP-2 and AP-3	(26–28)
P24 (Capsid)	Forms a cone-shaped shell that encapsulates the RNA-nucleocapsid complex and mediates its delivery into the host nucleus	Virion and host cell cytoplasm	Cyclophilin A, NUP358, NUP153, CPSF6, PIN1, TRIM5alpha, NUP98	(29–34)
P2 (Spacer Peptide 1)	Spacer peptide	Virion and host cell cytoplasm	No known interactions	
P7 (Nucleocapsid)	Encapsulates and protects viral genomic RNA and enhances various steps in virion reverse transcription	Virion, host cell cytoplasm and host nucleus	Alix, STAU1, ABCE1, EAP30, ESCRT-II, DHX9	(35–39)
P1 (Spacer Peptide 2)	Spacer peptide	Virion	No known interactions	
P6	Facilitates ESCRT-dependent virus budding and mediates Vpr incorporation into virions	Virion and host cell cytoplasm	ESCRT, Tsg101, Alix	(25,40,41)
Nef	Mediates MHC-I and CD4 down-regulation and prevents apoptosis	Cell plasma membrane and host cell cytoplasm	CD4, AP1M1, AP2M1, MHC-I, ASK1, Alix, ACOT8, PACS-1, PACS-2, PAK2, LCK, HCK, ABCA1, Calnexin, Catenin beta-1	(42–56)
Gag-Pol	Mediates essential virion assembly and the release of protease from Pol triggers virion maturation	Virion, host cell cytoplasm, cell plasma membrane and late endosomes/multivesicular bodies	Interaction experiments performed for Gag	
P15 (Protease)	Initiates virion maturation	Virion and host cell cytoplasm	N/A	
P51 (Reverse Transcriptase)	Converts viral ssRNA into dsDNA for its subsequent integration into the host genome	Virion, host cell cytoplasm and nucleus	N/A	
P15 (RNase H)	Removes the RNA template strand from the RNA/DNA duplex	Virion	N/A	
P31 (Integrase)	Catalyzes viral DNA integration into the host genome	Virion, host cell nucleus and cytoplasm	LEDGF/p75, INI1/SMARCB1, Importin alpha 3, Importin 7, UNG, Transportin-3, NUP153, NUP62, Gemin2	(57–65)
P19 (Rev)	Binds to the *Rev Response Element* (RRE) to facilitate the nuclear export of unspliced or incompletely spliced viral RNAs to the cytoplasm	Host cell nucleus/nucleolus and cytoplasm	CRM1/XPO1, Importin-beta1, B23, DDX3X, Sam68, DDX1, DDX5, DDX24	(14–20,66)
Tat (P14/P16)	Binds to the *transactivating responsive sequence* (TAR) RNA element to recruit certain host proteins and promote HIV-1 transcription	Host cell nucleus/nucleolus, extracellular regions and host cytoplasm	Cyclin T1, CDK9, P300, CBP, Sp1, DDX3X, NAP1L1, INI1/SMARCB1, MED14, TIP110, SMARCA4, Importin beta 1, NFAT1	(67–77)
P23 (Vif)	Counteracts the innate antiviral activity of host cytidine deaminase nucleic editing enzymes	Virion, host cell cytoplasm and membrane	APOBEC3G, APOBEC3F, APOBEC3D, APOBEC3H, ELOB, ELOC, CUL5, RBX1, CBF-beta	(78–85)
Vpr (p10/p12)	Plays a role in the nuclear entry of the viral cDNA genome and degrades host Uracil-DNA glycosylase. Also induces host cell G2 arrest	Virion and host cell nucleus	UNG, VPRBP/DCAF1, Importin alpha 1, RAD23A	(86–89)
P16 (Vpu)	Induces the down-regulation of CD4 and promotes progeny virion release by antagonizing host tetherin/BST2	Virion, host cell cytoplasm and membrane	CD4, βTrCp, NTB-A, CD155, tetherin/BST2, AP1M1	(90–95)

### (iii) Genomic location and protein sequence

**Figure 3.**
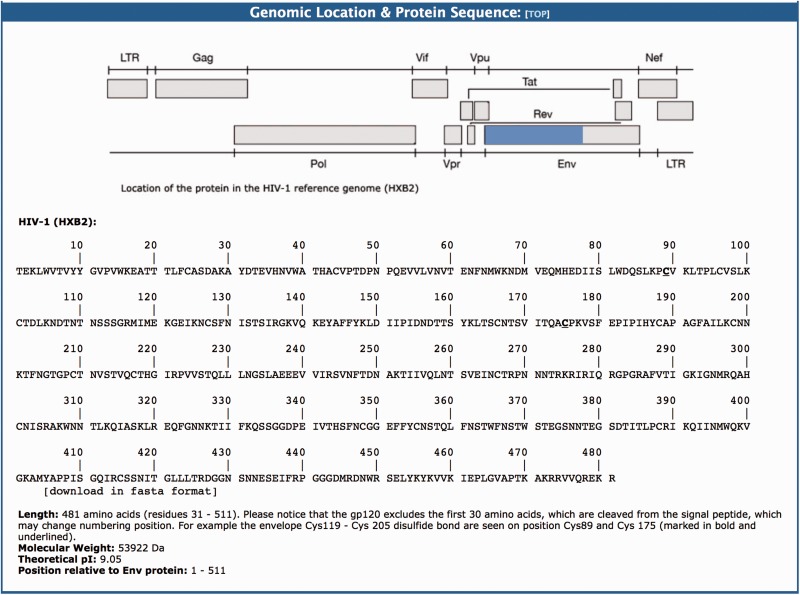
The genomic location and protein sequence section of the BioAfrica Proteome Resource Env gp120 protein.

This section provides a graphical representation of the location of the HIV-1 protein sequence in question relative to the HIV-1_HXB2_ reference genome (96). This is followed by the amino acid sequence data (FASTA format). For example, the gp120 is a protein that contains 481 amino acids with a molecular weight of 53 922 Da and theoretical PI of 9.05 (Figure 3). This protein is formed after a 30 amino acid signal peptide is cleaved from the amino terminal part of the ENV protein.

### (iv) Protein domains/folds/motifs

**Figure 4.**
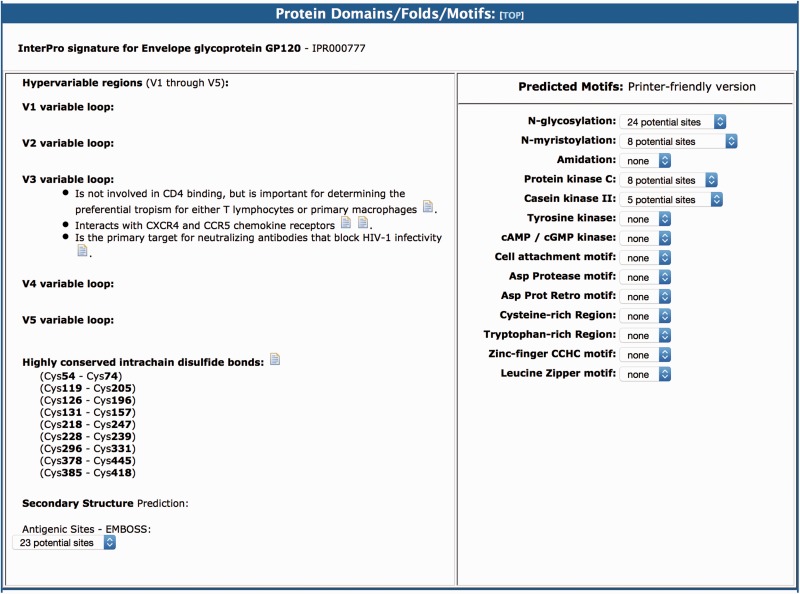
The protein domains/folds/motifs section of the BioAfrica HIV-1 Proteome Resource, exemplified by the gp120.

As in the original version of the BioAfrica HIV-1 Proteomics Resource, the section on protein domains/folds/motifs includes information about the predicted motifs and structure of the protein as well as the protein functional domains (1). For example, the gp120 had five variable loops (V1–V5). The V3 loop interacts with CXCR4 and CCR5 chemokine receptors and it is important for determining the preferential tropism for either T lymphocytes or primary macrophages (Figure 4). This section also includes information relating to protein secondary structure, low complexity regions, myristoylation, phosphorylation and glycosylation. For example, we list the Highly conserved intrachain disulfide bonds at cystein (Cys) Cys54–Cys74, Cys119–Cys205, Cys126–Cys196, etc. (http://bioafri ca.net/proteomics/ENV-GP120pro t.html).

### (V) HIV ARVs and drug resistance mutations

The ARVs and Drug Resistance Mutations section is a new development to the BioAfrica Proteomics Resource. The section includes all ARVs targeting an HIV protein. These include protease (www.bioafrica.net/proteomics/POL-PRprot.html), reverse transcriptase (http://www.bioafrica.net/proteomics/POL-RTprot.html), integrase (http://www.bioafrica.net/proteomics/POL-INprot.html) and envelope gp120/gp41 proteins (http://www.bioafrica.net/proteomics/ENVprot.html). For each of these protein pages, there is an additional section that provides users with an overview of the current ARVs. Information in this section also includes descriptions of the type and position of the mutation and relevant publications. Using Env as an example, there is only one fusion inhibitor that has been approved for HIV treatment, namely Enfuvirtide. This 36 amino acid polypeptide binds to the heptad repeat (HR) regions of the gp41 viral protein and engages in a coil-coil interaction. This interaction inhibits the fusion of viral and cellular membranes and thus prevents the entry and infection of HIV. There are six drug resistance mutations (positions 36–38, 40, 42–43) that have been well described in the literature (97) (Table 2). A further two potential mutations have been identified but need to be tested phenotypically. In addition to Env, there are ARVs and drug resistance sections on the reverse transcriptase, protease and integrase webpages. For example, the K103R mutation on the reverse transcriptase affects the ARVs Nevirapine (NVP), Delavirdine (DLV) and Efavirenz (EFV) and reduces the virus susceptibility to these drugs (7,13). Furthermore, when in combination with the V179D mutation, K103R mutants can decrease HIV susceptibility to NVP, DLV and EFV by 15-fold (99). All drug resistance mutations are based on the HIV-1 subtype B reference sequence (HXB2), however, we also link from the resource recent reviews that add information related to drug resistance to HIV-1 subtype C, which is the most prevalent HIV-1 strain in the world.

**Table 2.** The HIV ARVs and drug resistance mutations section of the BioAfrica HIV-1 Proteome Resource, shown by the Env polyprotein page (http://bioafrica.net/proteomics/ENVprot.html)

Protein position	Mutation	Additional information	Drugs affected	**Reference**
G36	D, E, V, S	G36D/E mutations are associated with a large decrease in Enfuviritide susceptibility (>10-fold)	Enfuvirtide	(97)
I37	V		Enfuvirtide	(97)
V38	E, A, M, G	V38E/A mutations are associated with a large decrease in Enfuviritide susceptibility (>10-fold)	Enfuvirtide	(97)
Q40	H	Mutation is associated with a large decrease in Enfuviritide susceptibility (>10-fold)	Enfuvirtide	(97)
N42	T, S	N42S occurs in ∼15% of viruses from Enfuviritide-naive patients and does not decrease drug susceptibility	Enfuvirtide	(97,98)
N43	D, K, S	N43D mutation is associated with a large decrease in Enfuviritide susceptibility (>10-fold)	Enfuvirtide	(97)
L44	M		Enfuvirtide	(97)
L45	M		Enfuvirtide	(97)

### (vi) Primary and secondary database entries

**Figure 5.**
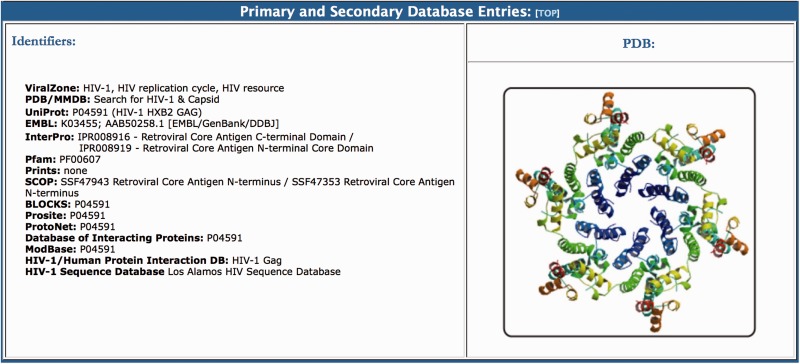
The primary and secondary database entries section of the BioAfrica HIV-1 Proteome Resource, seen on the Gag capsid protein.

The primary and secondary database entries section lists links to relevant online resources containing information about different aspects of the virus protein (Figure 5). Options include specific databases that provide users with sequence, function and protein-protein interaction data for each HIV-1 protein, as well as protein family annotations and post-translational modification information. A graphical representation (PDB format) of the protein is also provided in this section with links to the protein data bank entry. All of the proteome pages end with a list of the referenced articles, which are linked to PubMed. For example, for gp120 we provide 19 key references that were used in the curation process. The citations normally start with a link to the Los Alamos HIV Database Compendium and to the Retroviruses book, which are online accessible resources that contain detailed information about each protein. This is followed by the original publication on the function of each protein, which in the case of the envelope, is a Nature publication from 1988 that describes how a glycoprotein of HIV-1 binds to the immunoglobulin-like domain of CD4 (100).

## Discussion

Upgrading BioAfrica in collaboration with ViralZone and with the use of SwissProt curation methods was a time consuming but worthwhile undertaking. The process consisted of reading hundreds of manuscripts to critically review experimental and predicted data for each HIV protein as well as host proteins that interact with HIV. Curation included extracting and structuring information from the literature, manually verifying results from computational analyses and mining large-scale protein datasets. The process involved collaboration with professional curators from Switzerland and the training of South African researchers in biocuration. Furthermore, it provided synergy between BioAfrica and ViralZone information, which will allow users to access high-quality information that is available in two popular protein curation resources.

Prior to the upgrade of BioAfrica, the majority of resources only provided users with information about the virus or the host proteins. In addition, no resource linked this information to ARVs and drug resistance mutations. Our online resource provides comprehensive detail about various aspects of each HIV-1 gene product. It now includes information about protein isoforms, localization, function, sequence data (based on the HIV-1 reference sequence HXB2), protein domains/folds/motifs and host and virus protein-protein interactions. We believe that the easy access to well curated and current information will advance HIV drug resistance and HIV vaccine research and will provide a better understanding of the interaction between the host and the virus.

## Supplementary data

Supplementary data are available at *Database* Online. 

## Funding

the Swiss South African Joint Research Programme (SSJRP) research grant entitled "Swiss Prot / South Africa: Protein Bioinformatics Resource Development for Important Health- related Pathogens. The Swiss Federal Government through the State Secretariat for Education, Research and Innovation. Flagship grant from the Medical Research Council (MRC) of the Republic of South Africa (MRC-RFA-UFSP-01-2013/UKZN HIVEPI) the Wellcome Trust (082384/Z/07/Z) a Royal Society Newton Advanced Fellowship (T de Oliveira).

*Conflict of interest*. None declared.

## Supplementary Material

Supplementary Data
