# Supplementary material for: Improving HIV proteome annotation: new features of BioAfrica HIV Proteomics Resource
Source: Database (Oxford). 2016 Apr 16;2016:baw045. doi: 10.1093/database/baw045 (PMC4834208; doi:10.1093/database/baw045)
Supplement: Supplementary Data [file supp_2016_baw045_index.html]

Supplementary Data 

# Improving HIV proteome annotation: new features of BioAfrica HIV Proteomics Resource

## Supplementary Data

files

- Supplementary Data - pdf file
- Supplementary Data - pdf file
