# Supplementary material for: Improving HIV proteome annotation: new features of BioAfrica HIV Proteomics Resource
Source: Database (Oxford). 2016 Apr 16;2016:baw045. doi: 10.1093/database/baw045 (PMC4834208; doi:10.1093/database/baw045)
Supplement: Supplementary Data [file supp_baw045_Protease_NewBioAfrica.pdf]

New protease page
(http://www.bioafrica.net/proteomics/POL-PRprot.html)

BIOAFRICA BIOINFORMATICS & GENOMICS FOR HEALTH AND LIFE SCIENCES IN AFRICA

PR - Protease - Retropepsin

HIV **PR** is an aspartyl protease and is required for cleavage of Gag, Gag-Pol, Pol and Nef precursors and promotes virion maturation.

- p15 Protease GO!

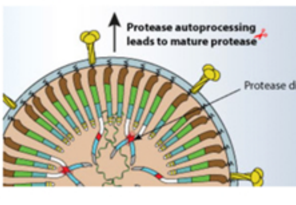

Key web links:
ViralZone: HIV-1, HIV replication cycle, HIV resource
PDB: 1AAQ (HIV-1 Protease)
UniProt: P04585 (HIV-1 HXB2 POL)
Chime Tutorial: Online Macromolecular Museum
HIV-1/Human Protein Interaction DB: HIV-1 Pol
Los Alamos HIV structure DB: Protease
EMBL: K03455 [EMBL/GenBank/DDBJ]

Isoforms:
- p15 (99 amino acids)

Cleavage sites:
- HIV-1 Pol **cleavage site** details

Function:
- Viral enzyme that initiates virion maturation.
- Shortly after the budding of the virion from the plasma membrane, in a process called maturation, the dimeric aspartyl **protease** cleaves **Gag** polyprotein, **Gag-pol** polyprotein and **Nef** precursors to give rise to the mature proteins.
- Hydrolyses host *EIF4GI* and *PABP1* in order to shut off the capped cellular mRNA translation to ensure maximal viral gene expression while evading host immune response.

Localization:
- Virion
- Host cell cytoplasm

Additional Information:
- Late timing of expression.
- **Protease** is an *aspartyl protease*.
- **Protease** functions while in a homodimer.

Protease Function & Host-Virus Protein Interactions: [TOP]

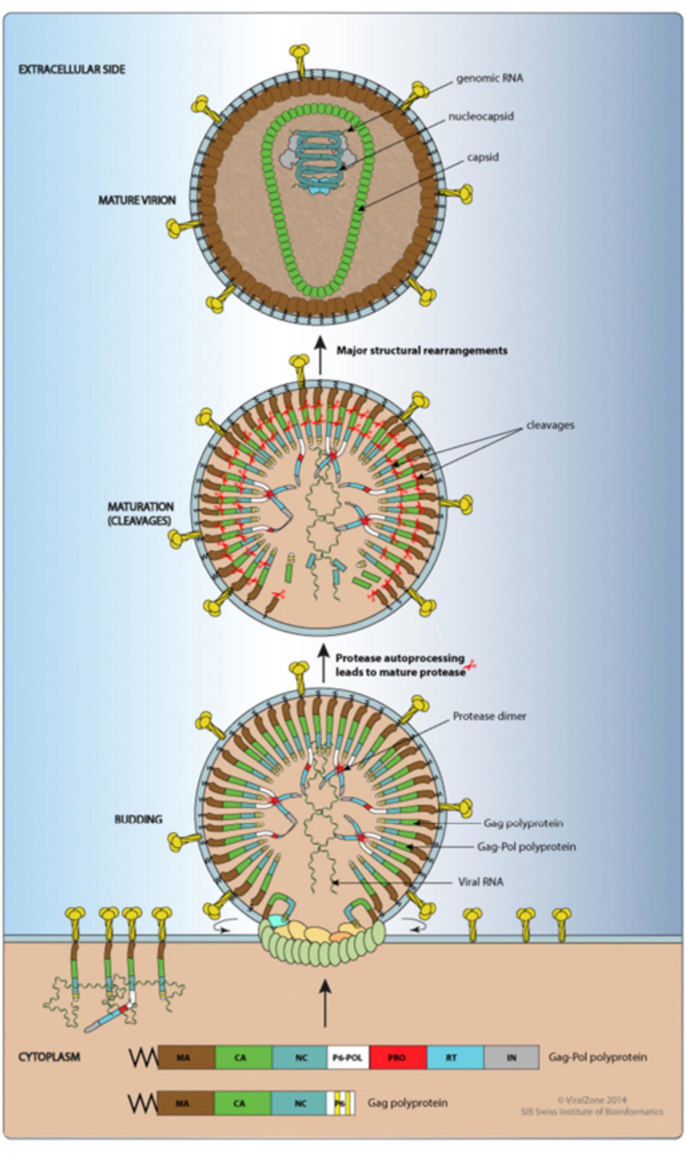

Interactions highlighted in the image:

- **Protease** cleaves the HIV **Gag** polyprotein.
- **Protease** cleaves the HIV **Gag-Pol** polyprotein.
- **Protease** cleaves the HIV **Nef protein**.

Potential interactions (not in the image):

- Host **EIF4G1** and **PABPC1** [see GUAVAh EIF4G1 interaction profile and PABPC1 interaction profile].

Genomic Location & Protein Sequence: [TOP]

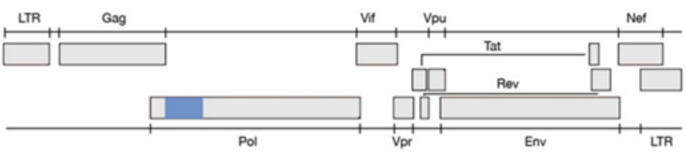

Location of the protein in the HIV-1 reference genome (HXB2)

HIV-1 (HXB2):

```
        10         20         30         40         50         60         70         80         90         99
         |          |          |          |          |          |          |          |          |          |
PQVTLWQRPL VTIKIGGQLK EALLDTGADD TVLEEMSLPG RWKPKMIGGI GGFIKVRQYD QILIEICGHK AIGTVLVGPT PVNIIGRNLL TQIGCTLNF
    [download in fasta format]
```

Length: 99 amino acids (residues 57 to 155)
Molecular Weight: 10779 Da
Theoretical pI: 8.83
Position relative to Pol protein: 57 - 155

Protein Domains/Folds/Motifs: [TOP]

InterPro signature for active site Aspartic Peptidase - IPR001969
InterPro signature for Retrovirus Peptidase A2A - IPR001995

Secondary Structure prediction:

Antigenic Sites - EMBOSS:
5 potential sites

Predicted Motifs: Printer-friendly version

Motif	
**N-glycosylation:**	none
**N-myristoylation:**	3 potential sites
**Amidation:**	none
**Protein kinase C:**	1 potential site
**Casein kinase II:**	2 potential sites
**Tyrosine kinase:**	none
**cAMP / cGMP kinase:**	none
**Cell attachment motif:**	none
**Asp Protease motif:**	1 potential site
**Asp Prot Retro motif:**	1 potential site
**Cysteine-rich Region:**	none
**Tryptophan-rich Region:**	none
**Zinc-finger CCHC motif:**	none
**Leucine Zipper motif:**	none

HIV Antiretrovirals and Drug Resistance Mutations: [TOP]

See Stanford University HIV Drug Resistance Database, which contains information different HIV-1 subtypes and CRFs 01 and 02

See a recent review on HIV-1 drug resistance and subtypes, with special focus on HIV-1 subtype C, the most prevalent HIV-1 strain in the world

See ViralZone HIV Drug Pages

Mechanism of Action:

Proteolytic cleavage of viral polypeptide precursors is absolutely required for the production of mature and infectious HIV virions.
Therefore, this step is an attractive target for antiviral therapy development.
The enzyme responsible for this process is the HIV **protease**, which is a member of the family of aspartyl proteases and possesses an aspartic acid at its active site (position 25).
The first **protease inhibitors** developed were active site inhibitors whose structure was based on natural substrates of **protease**.
These inhibitors contain a site that cannot be cleaved by **protease** and remain attached to the enzyme, preventing its proteolytic ability.
Unfortunately, due to the variable nature of the **protease** enzyme, drug resistance can occur.
Another treatment strategy involves the inhibition of **protease** dimerization.
For an enzyme to be active it must be folded as a dimer; therefore these **protease inhibitors** target the anti-parallel beta-barral region that is formed by N- and C-terminal parts of each monomer to prevent dimerization.
Currently ten FDA approved **protease inhibitors** (PIs) are available, including Tripanavir (TPV), which inhibits both protease dimerization and enzymatic activity.

Drug Resistance Mutations:

PI:

Position	Mutation	Additional Information	Drugs Affected	Reference
L23	I		NFV	
L24	I		ATV/r, FPV/r, IDV/r, LPV/r, NFV, SQV/r	
D30	N		NFV	
V32	I	Common mutation that develops in patients developing virologic failure while on APV or FPV treatment regimens. These cause decreases in FPV susceptibility.	DRV/r, FPV/r, IDV/r, LPV/r	
L33	F	Accessory mutation associated with decreased FPV susceptibility.	ATV/r, DRV/r, FPV/r, LPV/r, NFV, TPV/r	
M46	I, L		ATV/r, FPV/r, IDV/r, LPV/r, NFV, TPV/r	
I47	V	Common mutation that develops in patients developing virologic failure while on APV or FPV treatment regimens. These cause decreases in FPV susceptibility.	ATV/r, DRV/r, FPV/r, IDV/r, LPV/r, NFV, TPV/r	
I47	A		DRV/r, FPV/r, LPV/r	
G48	V, M		ATV/r, LPV/r, NFV, SQV/r	
I50	L	Hypersusceptibility mutation- increases susceptibility to all PIs except ATV/r.	ATV/r	
I50	V	Hypersusceptibility mutation- increases susceptibility to TPV/r. Common mutation that develops in patients developing virologic failure while on APV or FPV treatment regimens. These cause decreases in FPV susceptibility.	DRV/r, FPV/r, LPV/r	R Elston, HIV Drug Resistance Workshop (2006)
F53	L		ATV/r, IDV/r, NFV, SQV/r	
I54	V, T, A, L, M	Hypersusceptibility mutation I54L- increases susceptibility to TPV/r. Common mutations (I54M/L) that develop in patients developing virologic failure while on APV or FPV treatment regimens. These cause decreases in FPV susceptibility.	ATV/r, FPV/r, IDV/r, LPV/r, NFV, SQV/r	R Elston, HIV Drug Resistance Workshop (2006)
I54	L, M	Hypersusceptibility mutation I54L- increases susceptibility to TPV/r.	DRV/r	R Elston, HIV Drug Resistance Workshop (2006)
I54	V, A, M		TPV/r	
G73	S, T		ATV/r, DRV/r, FPV/r, IDV/r, NFV, SQV/r	
L76	V	Hypersusceptibility mutation- increases susceptibility to ATV, SQV, TPV/r.	DRV/r, FPV/r, IDV/r, LPV/r	S Mueller, HIV Drug Resistance Workshop (2004)
V82	A, T, F, S		ATV/r, FPV/r, IDV/r, LPV/r, NFV	
V82	A, T		SQV/r	
V82	A, T, F, S, L		TPV/r	
I84	V, A, C	Common mutation that develops in patients developing virologic failure while on APV or FPV treatment regimens. These cause decreases in FPV susceptibility.	ATV/r, DRV/r, FPV/r, IDV/r, LPV/r, NFV, SQV/r, TPV/r	
N88	D		ATV/r, NFV	
N88	S	Hypersusceptibility mutation- increases susceptibility to FPV/r.	ATV/r, IDV/r, NFV, SQV/r	
L90	M		ATV/r, DRV/r, FPV/r, IDV/r, LPV/r, NFV, SQV/r, TPV/r	

Primary and Secondary Database Entries: [TOP]

Identifiers:

ViralZone: HIV-1, HIV replication cycle, HIV resource
PDB/MMDB: Search for HIV-1 & Protease
UniProt: P04585 (HIV-1 HXB2 POL)
EC: 3.4.23.16
EMBL: K03455; AAB50259.1 [EMBL/GenBank/DDBJ]
MEROPS: A02.001
InterPro: IPR001969 - Eukaryotic/viral aspartic protease active site
IPR001995 - Retroviral Aspartic Protease family
Pfam: PF00077
Prints: none
SCOP: SSF50630 Acid protease
BLOCKS: P04585
Prosite: P04585
ProtoNet: P04585
Database of Interacting Proteins: P04585
ModBase: P04585
HIV-1/Human Protein Interaction DB: HIV-1 Pol
HIV-1 Sequence Database Los Alamos HIV Sequence Database

PDB:

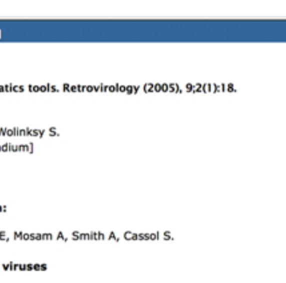

Reviews and References: [TOP]

Cite the resource by citing the following paper:
Doherty R et al. BioAfrica's HIV-1 Proteomics Resource: Combining protein data with bioinformatics tools. Retrovirology (2005), 9;2(1):18.

1 - **HIV Sequence Compendium 2000**
Kuiken CL, Foley B, Hahn B, Korber B, Marx PA, McCutchan F, Mellors JW, Mullins JI, Sodroski J, Wolinksy S.
Theoretical Biol. & Biophys. Group, Los Alamos Nat Lab, LA-UR 01-3860 [Read it online: Compendium]
2 - **Retroviruses**
Coffin JM, Hughes SH, Varmus HE.
CD-ROM ed. (2002) Cold Spring Harbor Laboratory Press [Read it online: NCBI Bookshelf]
3 - **Molecular Characteristics of HIV-1 Subtype C Viruses from KwaZulu-Natal, South Africa: Implications for Vaccine and Antiretroviral Control Strategies.**
Gordon M, De Oliveira T, Bishop K, Coovadia HM, Madurai L, Engelbrecht S, Janse van Rensburg E, Mosam A, Smith A, Cassol S.
Journal of Virology 77(4): 2587-2599 (2003) [pubmed: 12551997]
4 - **An inhibitor of the protease blocks maturation of human and simian immunodeficiency viruses and spread of infection.**
Ashorn P, McQuade TJ, Thaisrivongs S.
Proc Natl Acad Sci USA 87: 7472-7476 (1990) [pubmed: 2217178]
5 - **Crystal structure of a retroviral protease proves relationship to aspartic protease family.**
Miller M, Jaskolski M, Rao JK.
Nature 337: 576-579 (1989) [pubmed: 2536902]
6 - **Three-dimensional structure of aspartyl protease from human immunodeficiency virus HIV-1.**
Navia MA, Fitzgerald PM, McKeever BM.
Nature 337: 615-620 (1989) [pubmed: 2645523]
7 - [HIV Protease Drug Design - Review] **Aspartic proteinases in disease: a structural perspective.**
Cooper JB.
Curr Drug Targets 3(2):155-173 (2002) [pubmed: 11958298]
8 - [Website] **HIV Drug Resistance Database**
Stanford
Website: http://hivdb.stanford.edu
9 - [Website] **HIV Drug Resistance Database**
Los Alamos National Labs
Website: http://www.hiv.lanl.gov/content/sequence/RESDB/
10 - **HIVdb: a database of the structures of human immunodeficiency virus protease.**
Vondrasek J, Wlodawer A.
Proteins 49(4):429-31 (2002) [pubmed: 12402352]
11 - **Kinetic characterization of the critical step in HIV-1 protease maturation.**
Sadiq SK, No� F, De Fabritiis G.
Proc Natl Acad Sci U S A. 109(50):20449-54 (2012) [pubmed: 23184967]
12 - **F99 is critical for dimerization and activation of South African HIV-1 subtype C protease.**
Naicker P, Seele P, Dirr HW, Sayed Y.
Protein J. 32(7):560-7 (2013) [pubmed: 24132393]
13 - **HIV- 1 protease inhibits Cap- and poly(A)-dependent translation upon eIF4GI and PABP cleavage.**
Castell� A, Franco D, Moral-L�pez P, Berlanga JJ, Alvarez E, Wimmer E, Carrasco L.
PLoS One. 4(11):e7997 (2009) [pubmed: 19956697]
14 - **Protease inhibitors as antiviral agents.**
Patick AK, Potts KE.
Clin Microbiol Rev. 11(4):614-27 (1998) [pubmed: 9767059]
15 - **Drug resistance mutations for surveillance of transmitted HIV-1 drug-resistance: 2009 update.**
Bennett DE, Camacho RJ, Otelea D, Kuritzkes DR, Fleury H, Kiuchi M, Heneine W, Kantor R, Jordan MR, Schapiro JM, Vandamme AM, Sandstrom P, Boucher CA, van de Vijver D, Rhee SY, Liu TF, Pillay D, Shafer RW.
PLoS One 4(3):e4724 (2009) [pubmed: 19266092]
16 - **Identification of I50L as the signature atazanavir (ATV)-resistance mutation in treatment-naive HIV-1-infected patients receiving ATV-containing regimens.**
Colonno R, Rose R, McLaren C, Thiry A, Parkin N, Friborg J.
J Infect Dis. 189(10):1802-10 (2004) [pubmed: 15122516]
17 - **A mutation in human immunodeficiency virus type 1 protease, N88S, that causes in vitro hypersensitivity to amprenavir.**
Ziermann R, Limoli K, Das K, Arnold E, Petropoulos CJ, Parkin NT.
J Virol. 74(9):4414-9 (2000) [pubmed: 10756056]
18 - **Clinically validated mutation scores for HIV-1 resistance to fosamprenavir/ritonavir.**
Masquelier B, Assoumou KL, Descamps D, Bocket L, Cottalorda J, Ruffault A, Marcelin AG, Morand-Joubert L, Tamalet C, Charpentier C, Peytavin G, Antoun Z, Brun-V�zinet F, Costagliola D; ANRS Resistance Study Group.
J Antimicrob Chemother. 61(6):1362-8 (2008) [pubmed: 18390885]
19 - **Prediction of HIV-1 drug susceptibility phenotype from the viral genotype using linear regression modeling.**
Vermeiren H, Van Craenenbroeck E, Alen P, Bacheler L, Picchio G, Lecocq P; Virco Clinical Response Collaborative Team.
J Virol Methods 145(1):47-55 (2007) [pubmed: 17574687]
20 - **HIV-1 reverse transcriptase and protease resistance mutations selected during 16-72 weeks of therapy in isolates from antiretroviral therapy-experienced patients receiving abacavir/efavirenz/amprenavir in the CNA2007 study.**
Ait-Khaled M, Rakik A, Griffin P, Stone C, Richards N, Thomas D, Falloon J, Tisdale M; CNA2007 International Study Team.
Antivir Ther. 8(2):111-20 (2003) [pubmed: 12741623]
21 - **HIV protease mutations associated with amprenavir resistance during salvage therapy: importance of I54M.**
Murphy MD, Marousek GI, Chou S.
J Clin Virol. 30(1):62-7 (2004) [pubmed: 15072756]
22 - **Prevalence and impact of HIV-1 protease codon 33 mutations and polymorphisms in treatment-naive and treatment-experienced patients.**
Kozal MJ, Hullsiek KH, Leduc R, Novak RM, MacArthur RD, Lawrence J, Baxter JD; Terry Beirn Community Programs for Clinical Research on AIDS (CPCRA).
Antivir Ther. 11(4):457-63 (2006) [pubmed: 16856619]

Thanks to ViralZone at the Swiss Institute of Bioinformatics (SIB) for allowing the use of their images on bioafrica.net.
Page last updated by Megan Druce, Paula Sommer and Tulio de Oliveira (UKZN/Africa Centre) & Philippe Le Mercier, Chantal Hulo and Patrick Masson (SIB/ViralZone).
