# Supplementary material for: Improving HIV proteome annotation: new features of BioAfrica HIV Proteomics Resource
Source: Database (Oxford). 2016 Apr 16;2016:baw045. doi: 10.1093/database/baw045 (PMC4834208; doi:10.1093/database/baw045)
Supplement: Supplementary Data [file supp_baw045_Protease_oldBioAfrica.pdf]

Old protease page
(http://www.bioafrica.net/proteomics2005/POL-PRprot.html)

bioafrica *HIV bioinformatics in Africa*

PR - Protease - Retropepsin

HIV **PR** is an aspartyl protease and is required for cleavage of Gag, Gag-Pol, Pol and Nef precursors.

- p15 Protease GO!

Reference Sequences GO!

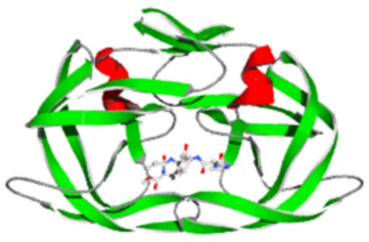

[animated gif: 320x240]

ViralZone:HIV-1
PDB: 1AAQ (HIV-1 Protease)
SwissProt: P04585 (HIV-1 HXB2 POL)
Chime Tutorial: Online Macromolecular Museum
Los Alamos HIV structure DB: Protease
EMBL: K03455 [EMBL/GenBank/DDBJ]
BioAfrica: Pol Protein Data Mining Tool

Isoforms:

- p15 (99 amino acids)

Cleavage site:

- HIV-1 Pol cleavage site details

Localization:

- Virion

Function:

- Viral enzyme
- Protease activity initiates virion maturation
- Processes Gag and Gag-pol polyproteins into mature chains

Additional Information:

- Protease is an aspartyl protease
- Protease functions while in a homodimer

Genomic Location: [TOP]

Reference Sequences:

HIV-1 (HXB2):

```
       10        20        30        40        50        60        70
        |         |         |         |         |         |         |
PQVTLWQRPL VTIKIGGQLK EALLDTGADD TVLEEMSLPG RWKPKMIGGI GGFIKVRQYD QILIEICGHK
       80        90        99
        |         |         |
AIGTVLVGPT PVNIIGRNLL TQIGCTLNF
    [download in fasta format]
```

Length: 99 amino acids (residues 57 to 155)
Molecular Weight: 10779 Da
Theoretical pI: 8.83

Protein Domains/Folds/Motifs: [TOP]

InterPro signature for active site Aspartic Peptidase - IPR001969
InterPro signature for Retrovirus Peptidase A2A - IPR001995
InterPro signature for Aspartic Peptidase Family - IPR009007

Secondary Structure prediction:

Antigenic Sites - EMBOSS:
5 potential sites

Predicted Motifs: Printer-friendly version

**N-glycosylation:**	none
**N-myristoylation:**	3 potential sites
**Amidation:**	none
**Protein kinase C:**	1 potential site
**Casein kinase II:**	2 potential sites
**Tyrosine kinase:**	none
**cAMP / cGMP kinase:**	none
**Cell attachment motif:**	none
**Asp Protease motif:**	1 potential site
**Asp Prot Retro motif:**	1 potential site
**Cysteine-rich Region:**	none
**Tryptophan-rich Region:**	none
**Zinc-finger CCHC motif:**	none
**Leucine Zipper motif:**	none

Protein-Protein Interactions: [TOP]

- Protease cleaves the HIV **Gag polyprotein**
- Protease cleaves the HIV **Gag-Pol polyprotein**
- Protease cleaves the HIV **Nef protein**

Primary and Secondary Database Entries: [TOP]

Identifiers:

ViralZone: HIV-1
PDB/MMDB: Search for HIV-1 & Protease
SwissProt: P04585 (HIV-1 HXB2 POL)
EC: 3.4.23.16
EMBL: K03455; AAB50259.1 [EMBL/GenBank/DDBJ]
PIR: UNKNOWN
HIV: K03455; UNKNOWN$HXB2
InterPro: IPR001969 / IPR001995
Pfam: PF00558;UNKNOWN
Prints: none
ProDom: PD186096 (1 - 16)
SCOP: SSF50630 Acid protease
BLOCKS: P04585
Prosite: P04585
ProtoNet: P04585
ProtoMap: P04585
PRESAGE: P04585
Database of Interacting Proteins: P04585
ModBase: P04585
Swiss-2DPAGE: 2D gel

BioAfrica Tools:
- Pol Protein Data Mining Tool provides real-time analysis of HIV-1 Pol isolates
- HIV Structure BLAST searches for similar HIV sequences that have known structures
- HIV Proteomics Resource contains protein sequence and structure analysis tools

Reviews and References: [TOP]

Cite the resource by citing the following paper:
Doherty R et al. BioAfrica's HIV-1 Proteomics Resource: Combining protein data with bioinformatics tools. Retrovirology (2005), 9;2(1):18.

1 - **HIV Sequence Compendium 2000**
Kuiken CL, Foley B, Hahn B, Korber B, Marx PA, McCutchan F, Mellors JW, Mullins JI, Sodroski J, Wolinksy S.
Theoretical Biol. & Biophys. Group, Los Alamos Nat Lab, LA-UR 01-3860 [Read it online: Compendium]

2 - **Retroviruses**
Coffin JM, Hughes SH, Varmus HE.
CD-ROM ed. (2002) Cold Spring Harbor Laboratory Press [Read it online: NCBI Bookshelf]

3 - **Molecular Characteristics of HIV-1 Subtype C Viruses from KwaZulu-Natal, South Africa: Implications for Vaccine and Antiretroviral Control Strategies.**
Gordon M, De Oliveira T, Bishop K, Coovadia HM, Madurai L, Engelbrecht S, Janse van Rensburg E, Mosam A, Smith A, Cassol S.
Journal of Virology 77(4): 2587-2599 (2003) [pubmed: 12551997]

4 - **An inhibitor of the protease blocks maturation of human and simian immunodeficiency viruses and spread of infection.**
Ashorn P, McQuade TJ, Thaisrivongs S.
Proc Natl Acad Sci USA 87: 7472-7476 (1990) [pubmed: 2217178]

5 - **Crystal structure of a retroviral protease proves relationship to aspartic protease family.**
Miller M, Jaskolski M, Rao JK.
Nature 337: 576-579 (1989) [pubmed: 2536902]

6 - **Three-dimensional structure of aspartyl protease from human immunodeficiency virus HIV-1.**
Navia MA, Fitzgerald PM, McKeever BM.
Nature 337: 615-620 (1989) [pubmed: 2645523]

7 - **[HIV Protease Drug Design - Review] Aspartic proteinases in disease: a structural perspective.**
Cooper JB.
Curr Drug Targets 3(2):155-173 (2002) [pubmed: 11958298]

8 - **[Website] HIV Drug Resistance Database**
Stanford
Website: http://hivdb.stanford.edu

9 - **[Website] HIV Drug Resistance Database**
Los Alamos National Labs
Website: http://resdb.lanl.gov/Resist_DB/default.htm

10 - **[Website] HIVdb: a database of the structures of human immunodeficiency virus protease.**
NIST
Website: http://srdata.nist.gov/hivdb/
